# Beyond the Gene

**DOI:** 10.1371/journal.pone.0001231

**Published:** 2007-11-28

**Authors:** Evelyn Fox Keller, David Harel

**Affiliations:** 1 Program in Science, Technology, and Society, Massachusetts Institute of Technology, Cambridge, Massachusetts, United States of America; 2 Department of Computer Science and Applied Mathematics, Weizmann Institute of Science, Rehovot, Israel; Baylor College of Medicine, United States of America

## Abstract

This paper is a response to the increasing difficulty biologists find in agreeing upon a definition of the gene, and indeed, the increasing disarray in which that concept finds itself. After briefly reviewing these problems, we propose an alternative to both the concept and the word *gene*—an alternative that, like the gene, is intended to capture the essence of inheritance, but which is both richer and more expressive. It is also clearer in its separation of what the organism statically is (what it tangibly inherits) and what it dynamically does (its functionality and behavior). Our proposal of a *genetic functor*, or *genitor*, is a sweeping extension of the classical genotype/phenotype paradigm, yet it appears to be faithful to the findings of contemporary biology, encompassing many of the recently emerging—and surprisingly complex—links between structure and functionality.

## Introduction

“...we cannot improve the language of any science without at the same time improving the science itself; neither can we, on the other hand, improve a science, without improving the language or nomenclature which belongs to it.”(Lavoisier, 1790, quoted in Goldenfeld and Woese [1])

### Background

Many people have recently argued that, after a century of extraordinary productivity, the concept of the gene has begun to outlive its usefulness. Among the reasons generally given is the great difficulty that we encounter today in trying to reach any sort of consensus about what a gene actually is. Here is what we read in a recent issue of *Nature*:

“Where the meaning of most four-letter words is all too clear, that of gene is not. The more expert scientists become in molecular genetics, the less easy it is to be sure about what, if anything, a gene actually is.”Helen Pearson [2]

Indeed, in recent years it has become even more difficult to get a group of scientists engaged in one aspect of genetics or another to agree on a definition. For example, two days of intense debate among 25 bioinformatics researchers produced the following definition:

“A gene is: ‘a locatable region of genomic sequence, corresponding to a unit of inheritance, which is associated with regulatory regions, transcribed regions and/or other functional sequence regions’.”*Sequence Ontology Consortium*, 2006

Clear? Well, not exactly. But still, these scientists seem to agree that a gene is “a locatable region of genomic sequence”, which might be because they are all working in bioinformatics, i.e., on the analysis of sequence information. Susan Lindquist, former Director of the Whitehead Institute, is a more conventional experimental geneticist, working with organisms, and here is what she has to say:

“Most people think of genetics as being only about DNA. But genetics is about the inheritance of traits. While most traits are inherited through transmission of DNA, the traits we study are inherited through proteins. Thus, these proteins can be every bit as much a genetic element as DNA. After all, they are heritable entities that span generations.”Susan Lindquist [3]

Lindquist challenges the material basis of heredity, but perhaps even more than the bioinformatics people, she remains happy with the notion of “genetic elements”, of heritable entities. This begs the question of what *is* a genetic element. Even confining ourselves to DNA, we have begun to encounter enormous difficulties in defining genetic elements of any kind. In 2000, one of the greatest ambiguities of the term gene came from the process of alternative splicing—that is, the process of rearranging the transcripts of a definite number of exons (protein-coding units) found within a particular region of the DNA. The transcript that coded for a particular protein appeared only in the form of RNA, after extensive editing and splicing, but many—even thousands—of such transcripts (and hence proteins) could be formed from the same sequence of DNA. Today, such difficulties have exploded, and they have come to challenge the very idea of a discrete and particulate unit of inheritance. As Pearson writes, “The idea of genes as beads on a DNA string is fast fading.”

By far the most common association between DNA and the word *gene* remains that of protein-coding sequences. But even protein-coding sequences have no clear beginning or end. Pearson continues:

“Instead of discrete genes dutifully mass-producing identical RNA transcripts, a teeming mass of transcription converts many segments of the genome into multiple RNA ribbons of differing lengths. These ribbons can be generated from both strands of DNA, rather than from just one as was conventionally thought. Some of these transcripts come from regions of DNA previously identified as holding protein-coding genes. But many do not. [Furthermore,] many scientists are now starting to think that the descriptions of proteins encoded in DNA know no borders — that each sequence reaches into the next and beyond.”

Worse yet, exons, protein coding sequences, turn out to be a rather small part of inheritance, even on the level of DNA. Today we know that much more than DNA sequence is passed on from one generation to another, but even restricting ourselves to this DNA, which many would argue is the only truly tangible part of an organism that is actually passed down, we read that, at least for higher organisms, a mere 1–2% of the genome is spanned by protein-coding sequences. Thus, while the entire DNA is inherited, and indeed, most of it is transcribed, only a very small fraction is involved in making and maintaining an organism through the construction of proteins. What is the rest of the DNA for? Over the last 15 years or so, we have learned of an entirely new genre of function that DNA sequences can have. Indeed, small sequences of RNA transcripts—sequences that have nothing to do with coding but a great deal to do with regulation—are the hot new actors in molecular genetics. Sometimes these sequences are referred to as non-coding RNA (ncRNA) genes.

As Pearson observes, most geneticists are not trying to find a definition of the gene on which they can agree. Instead, they tend to use “less ambiguous words such as transcripts and exons. [And even] when it is used, the word ‘gene’ is frequently preceded by ‘protein coding’ or another descriptor.…. Some things,” she concludes, “are not best portrayed by a crude four-letter word.”

We can elaborate on this theme, noting if only briefly the enormous variety of entities to which the term gene is currently applied. We speak of house-keeping genes, structural genes, regulator genes, promoter genes, operator genes, coding genes, non-coding genes, micro RNA genes, nested genes, overlapping genes, spliced genes, dead genes, etc. As Sydney Brenner put it with his typical acerbity, “Old geneticists knew what they were talking about when they used the term ‘gene’, but it seems to have become corrupted by modern genomics to mean any piece of expressed sequence” [4]. In a similar vein, and expressing similar frustration, genomicists Michael Snyder and Mark Gerstein suggest that “it might be better to define a molecular parts list … rather than whole genes.” [5]

One of the difficulties with such observations is that, accurate as they might be, for practical purposes they tend to be purely negative. While recognizing that the concept of the gene cannot be dispensed with altogether, they offer no positive proposal for what might be put in its stead. Even when speaking, in a somewhat more positive note, of “the century beyond the gene” (see, e.g., Keller [6]), we have had little if anything to say about exactly what that might be. What is it that lies beyond the gene?

In an illuminating recent review, Kapranov, Willingham and Gingeras [7] survey a large amount of evidence pointing to the unexpectedly complex ways in which DNA works. These all go well beyond the notion of a gene as a piece of DNA whose function is to produce a protein. They include the use of common pieces of DNA for multiple, often quite different, purposes; the use of overlapping sequences in the same or in different functions; the importance of “long-range elements” in helping to bring about a function; head-to-head transcription on the one hand and far-away transcription brought together on the other; both sense and antisense transcription; both *cis* and *trans* mechanisms; and issues related to timing in the processes of transcription and translation. Several of these can be present simultaneously in the process of going from genotype to phenotype.

A typical example is given in Figure 3 of Kapranov et al [7, p. 418], which we reproduce below, together with its explanatory caption. It concerns two RNA-based signaling pathways (one of which is known and the other is realistic but as of yet hypothetical). While the reader is encouraged to take a closer look at the details there, we point out that one of the main actors (an miRNA) is encoded by a piece of DNA that is located physically within a protein-coding “host gene”, and after a nontrivial process of maturation, this actor can, among other things, influence the expression of its own host sequence—i.e., the stretch of DNA that shares the sequence that is responsible for its own existence.

**Figure 1:**
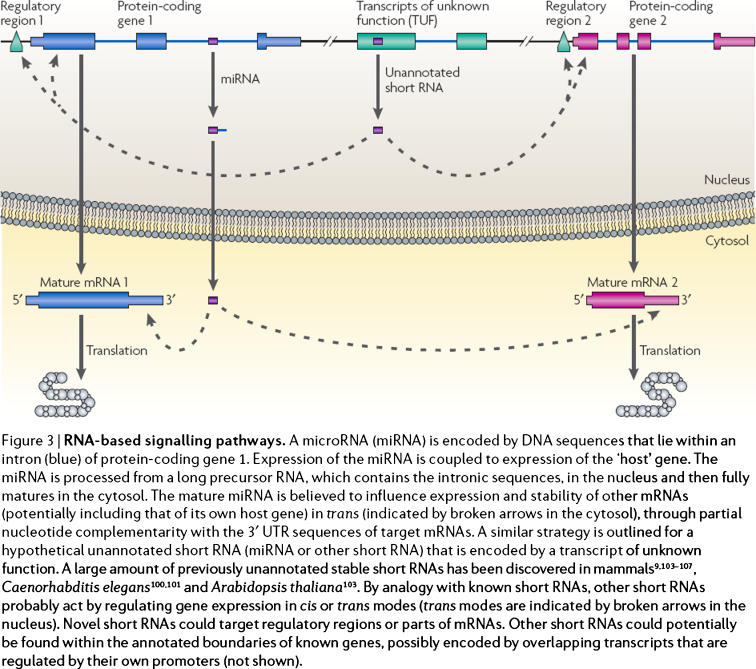
This is Fig. 3 from Kapranov, Willingham and Gingeras [7] reproduced verbatim, complete with its original caption. (Reprinted by permission from Macmillan Publishers Ltd: *Nature Reviews Genetics*.)

Kapranov et al write, “These observations suggest that genomic architecture is not colinear, but is instead interleaved and modular, and that the same genomic sequences are multifunctional” [7]. Their argument has now been strikingly confirmed by the first results of the ENCODE Project Consortium [8]. These findings are of immense interest, and dramatically underscore the need for new ways of thinking about DNA sequences, genomic organization, and their relationship to function. If we have learned nothing else, it is that that relation is far more complex than we had ever anticipated. Yet more recently, Gingeras [9] suggests that this increased complexity necessitates “a reconsideration of the definition of a gene and require[s] the use of an alternative term to help to define the fundamental operational unit that relates genomic sequences to phenotypes/function.”

### What we are trying to do

This paper, in which we seek to redress the deficits in the gene concept, is a response to the need that Gingeras identifies. More specifically, we would like to suggest as a replacement for that concept a framework that is concrete, yet both expansive and flexible, as well as rich in expressive power. One that is better grounded in biological findings than the gene has proven to be, that might prove a workable language for the century beyond the gene, but that nonetheless retains what has proven to be most valuable about that concept.

To this end, we start by shifting focus from the abstract concept of the gene to that very concrete molecule with which we have become so familiar, the DNA. But we do not invoke the DNA of old—not DNA as the master molecule that embodies the secret of life, that encodes and orchestrates the dance of life, but rather the far more interesting molecule that we have come to know as DNA today. True, this DNA is no longer the only important actor in development, in heredity, or in evolution—indeed, given the passivity of the role it plays in all its interactions, it may not even be appropriate to call it an actor at all. As Elizabeth Pennini puts it, we have learned that “this molecule can't dance without a team of choreographers”, that “it comes alive only when numerous proteins pull its ‘strings’” [10]. Nevertheless, DNA has revealed itself as a far richer resource both for the construction of the many kinds of actors that do animate the cell and for the articulation of arenas for action that were never imagined in the simplistic mantra of “DNA makes RNA, RNA makes protein, and proteins make us.”

Earlier, we somewhat derisively cited a definition of the gene offered by the Sequence Ontology Consortium, deriding it for being impossibly vague. Now however we want to take a lesson from that effort. The fact of the matter is that today one finds the word gene used with such a vast range of references that the search for a useful common denominator seems pretty hopeless. Yet, as the effort of those bioinformatics researchers indicates, there *is* a common denominator to many uses of that word, and even if it may seem hopeless to fit into the straight-jacket of the old concept of the gene, we do think that common denominator needs to be respected.

Accordingly, the first thing we'd like to do is to offer as a replacement for the gene a concept that is closely related, even if of a different kind, which we shall call the *dene*. Like the gene, our notion of dene is intended to capture the essence of genetic transmission, but, rather than being confined to denoting a discrete chunk of DNA, it is far richer and more expressive. A dene is, in fact, a general kind of *statement* about the DNA—what logicians call a predicate or a property. Denes can be used to represent vastly more intricate characteristics of the DNA sequence than the simple statement that it contains a particular subsequence. Also, even though we choose (for now) to focus on the material structure of DNA because of its obvious importance in heredity, it should be obvious that everything we say about DNA (and denes) would also apply to other inheritance systems. (For example, a heritable chromatin mark associated with a change in transcription patterns would reflect another kind of linkage between structure and function, and would require its own terminology. Also, we might consider a variety of epigenetic functors—or epigenitors—that would relate a corresponding variety of epigenetic structures to behavior.) Most important of all, the way we shall use the notion of a dene, and the context in which we will place it, permits a clear separation between what the organism is constituted of (and relatedly, what it has materially inherited) and what it dynamically does with this material inheritance (i.e., the associated functionality and behavior). For symmetry, we will refer to a statement about behavior as a *bene*. As with denes, our notion of bene will also be extremely rich, making it possible to express complex modal and temporal characteristics of the organism's behavior over time, characteristics that go far beyond simple statements about, e.g., protein synthesis or transcription.

Denes and benes are obviously linked, and we consider this fact to be at the heart of any discussion of inheritance and genetics. In fact, it makes little sense to specify a dene—i.e., to make statements about a DNA sequence—without identifying the behaviors with which that sequence has come to be associated. Accordingly, we propose to be explicit about this conjunction, and thus introduce as our main concept, the *genetic functor*, or the *genitor*. This we define as the logical relation that says: Whenever the organism is seen to have X, it does Y. Or to use our new terms, a genitor relates a particular dene to a particular bene, stating that whenever the organism's DNA is seen to satisfy the property expressed by the dene, it's behavior satisfies the property expressed by the bene.

We argue that the genetic functor is faithful to the findings of both classical and contemporary biology, and that it encompasses many of the recently emerging—and far more complex—links between structure and functionality. Indeed, our aim is to offer a reframing that is inspired–perhaps even demanded–by what biologists are already doing, and our hope is that the new term might just possibly enable current and future research to move forward more effectively.

But it is time to put some flesh on our proposal. We begin with a logical formulation, and proceed by elaborating this formulation in relation to specific examples.

## Analysis

### Denes, benes and genitors

We shall use O to denote an organism of a specified type (i.e., with specified genetic and behavioral properties). We might think of it as an individual living being (or plant, etc.) or as a *form* thereof, i.e., an appropriately defined collection of individuals. Syntactically, a *genetic functor*, or *genitor*, G is defined as a triple G = (O, D, B), which groups together the organism O with a *dene* D and a *bene* B. The former is a statement about O's DNA and the latter is a statement about O's behavior. Both of these will be described in more detail below, but for now it suffices to say that, semantically, the dene D is a truth-valued function of O's DNA sequence and the bene B is a truth valued function of O's temporal life-span. Thus, a dene can be viewed as relating to a snapshot, taken with a still camera, of the organism's most profound inherited artifact, and B can be viewed as relating to a movie, taken with a video camera, of the way the organism dynamically develops, lives, behaves, etc. A dene thus captures something tangible about what the organism inherently *is*, and a bene captures something about what it *does*, or what it is capable of doing, always of course in the context of its internal and external environment. As to the semantics of their combination, we say that the genitor G = (O, D, B) is *true*, or *satisfied*, or that it *holds*, when it is the case that if O's DNA satisfies D then its existence over time satisfies B.

The genetic functor framework lends itself nicely to concise ways of discussing many notions—conceptual, experimental or theoretical—that are related to genomics, functionality and inheritance. A genitor G = (O, D, B) might be known (or found) to be true, yet G′ = (O′, D, B), in which we have retained D and B but have replaced the organism O with some closely related organism O′, might very well be false. Similarly, keeping now O and B fixed, G might become false if we replace the dene D with a closely related D′ (expressing, e.g., a mutation in O's DNA). And, of course, the same goes for exploring the effect of the dene D on different behaviors B and B′.

So much for the general framework. What now about the components of a genetic functor, the denes and the benes? For a start, what kinds of things are we interested in saying about an organism's DNA, what are the properties of the DNA that functionally relate to the organism's behavior?

Before proceeding, however, we should make it clear that including the organism O itself in the definition of a genitor has profound implications. O comes complete with all of its other cell-based machinery: In addition to DNA, it has RNA, proteins, ribosomes and metabolites, as well as metabolic systems and macromolecular complexes in particular states. So that when we claim, or prove experimentally, that the genitor G = (O, D, B) is true, the fact that O exhibits behavior B in a particular environment if its DNA satisfies D takes on the richer meaning that B is indeed a result of its DNA having the property D, but only given that the machinery required for B's realization already exists in O's cells. Thus, by including O in the definition of a genitor, we are also including whatever devices O employs to make use of a particular property of DNA in order to actually bring about the behavior B. On the other hand, the very statement that a genitor (O, D, B) is true—especially if accompanied by another genitor (O, D′, B′), postulating that in the same organism, a different D′ causes a different behavior B′—allows us to separate out and make explicit the role of DNA in the form of the dene D, and connect it to the associated behavior B. Similarly, the behavior B in a genitor (O, D, B) is intended to encompass anything one wants to say about O's behavior, including elements influenced by its environmental dependency. And this too has profound implications for the expressivity and usefulness of genitors.

It will be noticed that between genes and denes lies a difference of only one letter, yet, we argue, the latter, as we use it, designates a concept on an altogether different logical level. The crucial question is, what can a dene D express? Later we take up the issue of formalizing a language for denes, but for now we shall discuss briefly what such a language should make possible. We can think of an *atomic property* of a DNA molecule as being the statement that it contains, somewhere, the subsequence X. This is close to what many people would take to be the essence of a classical gene. Now, by way of extending this, we want denes to be able to contain many such atomic statements, i.e., to refer to many subsequences of the DNA, and to allow these subsequences to combine in different ways. They can be ordered or not, overlapping or not, negated or not, iterated or not, appearing as is or in reverse, be part of implications or not, necessarily (or with high probability) appearing, being forbidden to appear, etc. In fact, we want denes to be atomic properties combinable by logical operators, ordering, probabilities and modalities.

Using S to denote the organism O's complete DNA sequence (thought of as having a start and an end), here are some abstract examples of denes:

- S contains X followed somewhere downstream (i.e., further along in the sequence) by Y, but Z does not appear anywhere in between them.
- If S contains XY (i.e., X and Y contiguously) and somewhere downstream it contains both Y and Z, in any order, then W must appear between the XY and the later appearing Y.
- S does not contain X = YZ, but if it contains W overlapping with at least 50% of an occurrence of Y, then it must contain both Y and Z, in that order.
- If, when “moving along” S we encounter a sequence that consists exactly of two appearances of X and one each of U, Y and Z, in any order, then the probability (over all instances of the organism in question) that Z is not last is less than 0.2.
- S contains some non-zero number of contiguous X's (this is often written X^+^) not followed by a Y.
- S does not satisfy D; i.e., the logical negation (or set-theoretic complement) of the property denoted by some given dene D.

As these examples show, a genitor's dene may refer to the organism's entire genome or to some part thereof whose boundaries may be fixed or variable; it may refer to contiguous parts of the genome or to a disjoint collection of parts; these parts may be asserted to *have* to occur, to *possibly* occur, or to be *forbidden* to occur; they may be required to occur with order or without, or with some partial order; they may be conditional or negated; and they may contain overlapping or iterations of subsequences. This is one of the manifestations of rich logical expressiveness. The statement a dene makes need not be a simple and direct property of the DNA sequence; it can refer to far more complicated characteristics thereof, yet it is still a property/predicate of the sequence. And for each sequence, the dene is, clearly and unambiguously, either true or false in the sense that the DNA either has the indicated property or not.

Note that the dene alone says nothing about function. It makes a statement about the DNA as a static entity, as a fixed sequence. It is the role of the bene B to specify the associated behavior, and together they form the genitor that expresses the functional relation between D and B. What is important to reemphasize here is that a dene's constitutive component elements are always to be found in the DNA; denes, one might say, are made of DNA. But since a dene is a statement *about* the DNA—a designated *property* of the DNA—it cannot itself be said to *reside* in the DNA.

Let us now turn to benes. The function of the property of the DNA to which a dene refers may be regulative, or constitutive, informing or informative. These parts may or may not be transcribed into RNA. Thus, e.g., a dene may comprise the specification for one or more proteins, or it may serve as template for the production (transcription) of an RNA molecule that has a purely regulative function. It also might designate a binding site for a protein or RNA molecule, or it may comprise sequences that influence (shape or inform) the 3D structure of the DNA, its mutability, the location of nucleosomes, or even certain aspects of post-transcriptional regulation. For the bene B of the genitor G = (O, D, B), almost anything goes. Anything the organism O does that is a manifestation—even if only very indirectly so—of the fact that its DNA possesses the characteristics defined by the dene D can be made part of the bene. This includes taking into account O's environment, its internal mechanisms, and so on. On the cellular level, for example, cell death, movement and proliferation are to be allowed in benes. Later we describe in some detail several nontrivial examples, mostly taken for contemporary research, and the B parts of these exemplify the variety of biological behavior and function that we expect benes to be able to capture.

In analogy with the earlier discussion on the expressive richness of denes, we should now elaborate upon the rich expressivity of benes. Again, we shall talk about languages and formalization later, but here we briefly discuss the required concepts. Recall that benes also describe properties, except that here these are not properties of a static sequence but of a dynamic movie-like capture of O's behavior over time. This too is a sequence, but not of nucleotides: it is a sequence consisting of *events* and *actions*, either internal to the organism or cell, or external to them; reflecting changes in state, structure, value, shape, potential, location, etc. Events are things that happen, occur, and actions are things that are done, carried out. The events and actions can be related to each other in a variety of ways—causal, temporal, modal, Boolean, stochastic, etc. They can also be related in various ways to the actual entities (e.g., mechanisms) residing in the organism O itself; they can be created or set off as a result of these mechanisms, or, conversely, they can serve as triggers or catalysts thereof.

The term used in computer science for this kind of sequence is a *trace*, and the kind of behavior a trace captures is called *reactivity*. The type of system all this reflects is called a *reactive system* [11]. In fact, biological systems have been likened to reactive systems on multiple levels of description, including the intra-cellular, the inter-cellular, and those of the organ and organism (Harel [12]). So what we want really is for a bene (or more precisely, for its semantics) to be a property of the reactive behavior of the organism O and of O's actual constituents, always, of course, with respect to its environment. Note that the organism will in general have many possible traces of behavior, which might be determined in a variety of ways, perhaps stochastically, perhaps as a result of internal or external (environmental) events.

To help make benes more manageable, and possibly more amenable to the process of expressing complex organism behaviors, it might be beneficial at a later stage to make the environment (E) and internal mechanisms (M) explicit. We could then define a bene as an expression of the form “if E and M then B”, where E would express the relevant statements about the environment and M the relevant internal mechanisms. B would then be the raw behavior that is implied by the dene D under the assumptions expressed in the E and the M. For simplicity of the exposition we have decided not to do so, despite the fact that it makes the concept of a bene rather all-encompassing and perhaps unduly vague.

Here now are some simple abstract examples of what we want benes to be capable of expressing. We don't refer to the traces explicitly, but implicitly, each bene asserting its truth for every possible trace of behavior. Note the similarity with the examples for denes given earlier:

- If event F occurs then sometime later action G will be carried out, but H will not occur anywhere in between them.
- If the environment does F than O will do G and H in any order, but within 3 minutes, with probability 0.8.
- Whenever F occurs and is followed by at least three occurrences of G all within 2 seconds, then H will not be done before F will have occurred at least twice more in the interim.
- O's trace of behavior must contain some non-zero number of occurrences of F followed by a G, but the probability that H will ever be carried out after G occurs is less than 0.05.
- If O has the mechanism M, then whenever F occurs, M will cause G to be carried out between 1 and 2 seconds later.
- S does not satisfy B; i.e., the logical negation of the property denoted by some given bene B.

Thus, here too, events and actions may be asserted to *have* to occur, to *possibly* occur, to be *forbidden* to occur; to occur with some time constraints or under some probability; to be the result or cause of some environmental event; or to be the outcome, generator, (or for that matter the destroyer) of some mechanism.

So these are our genetic functors—our genitors. They capture, we think, the essence of living phenomena. A genitor, with its dene and bene, connects the static with the dynamic. It carries no expectation that its truth can be predicted on the basis of purely structural information. Furthermore, the multiplicity of references attaching to denes (or to genitors) is no longer an embarrassment. Rather, such multiplicity is to be expected. Indeed, it is to be celebrated; it is an indication that the cell has learned new ways of making use of the same sequences. It is evidence of the *generativity* of DNA—in the sense, i.e., that a given molecule of DNA can be used by cells to generate more and more novelty. We have discovered some of these new ways, but are surely still in the dark about others.

Denes are in fact far more expansive than the nomenclature of genes in contemporary genomics; they include not only “any piece [or pieces] of expressed sequence”, but also sequences (or Boolean, modal and order-related combinations thereof) that have functional significance even without being expressed. They allow for the invention of new functions without the intermediary of transcripts or codes. In the world of genitors, anything goes; i.e., anything that turns out to serve a useful—or useable—function. Rather than a book of life, DNA is an entity capable of generating a “Library of Babel” of the sort that Jorge Luis Borges described, a “universe (which others call the Library)… composed of an indefinite and perhaps infinite number of hexagonal galleries”; a universe in which one can find no “catalogue of catalogues”; a library that “includes all verbal structures ... but not a single example of absolute nonsense”. “It suffices,” Borges tells us, “that a book be possible for it to exist. Only the impossible is excluded” [13]. For Borges, the meaning of ‘impossible’ is not specified, but for biological organisms it is: Impossible is what cannot be tolerated by natural selection.

### Some examples

It is time to give some concrete and nontrivial examples of denes, benes, and genitors.

▪ As we've already suggested, the entire genome of an organism can itself be considered a dene (or more accurately, the dene would simply state that the organism's genome is equal to a given sequence S). Without the genome, the organism would not exist; indeed, the entire range of development and behavior would be the associated bene, and the genitor would constitute the set of relations linking the two.

▪ The classical polypeptide coding unit found as a continuous stretch of DNA bounded by a stop and start codon (corresponding to Seymour Benzer's *cistron*) is easily captured by a dene. The corresponding bene would be the production of a polypeptide chain (including transcription and translation), and the genitor would then capture the link between the two.

▪ Alternative splicing: Here a dene refers to any set of mRNA transcripts sewn together to form a protein-coding unit. In general, the dene would not require these to be contiguous. Each such dene would be associated with the corresponding polypeptide, the genitor capturing the relation that specifies the components of the dene corresponding to the bene that specifies production of the polypeptide.

▪ A dene can be defined to capture an association of stretches of DNA (contiguous or not) transcribed into an RNA molecule involved in the regulation of transcription, translation, or post-translational events. Most famously, such denes would correspond to what are currently called non-coding RNA genes, defined as “genes for which RNA, rather than protein, is the functional end product” (Klein et al [14]). The idea of transcribed but untranslated genes is not new (recall genes for rRNA, tRNA); what is new is the extent of their role in regulation. Non-coding RNA genes may correspond to small or large RNA transcripts, but among the best understood are those frequently referred to as micro-RNA genes, stretches of DNA comprising approximately 21 nucleotides, the RNA product of which is involved in target gene regulation and epigenetic silencing (Hsu et al [15]), siRNA (also involved in gene silencing, and distinguishable from micro-RNA only by their biogenesis); stretches of DNA transcribed into small nucleolar RNAs (snoRNAs), stem-bulge RNAs (sbRNAs) and small nuclear-like RNAs (snlRNAs). Importantly, the sequences referred to by such denes are not spatially separable from those of other denes, and may even be located inside still others (see, for example, the discussion in Kapranov et al [7]). The range of behaviors (benes) associated with such denes is evidently very large, and the subject of much current research. In our terminology, defining the appropriate genitors that would relate such benes to particular denes in a clean and rigorous fashion is the crux of the problem.

▪ Nucleotide sequences that shape the 3D-d conformation of larger stretches of the DNA molecule can also be defined as denes. For example, it has been known for some time that the folding patterns of both DNA and RNA depend on nucleotide sequences; that palindromic sequences promote the formation of hair-pin structures in DNA. However, more recent work has shown that transcription rates are also informed by histone binding. And the placement of nucleosome structures, as well as the binding specificity of histones, is similarly informed by DNA sequence, with probable sites for histone binding and for bends in the DNA predicted by the presence of DNA motifs of approximately 150 base pairs (see, e.g., Richmond and Davey [16]). These motifs would easily qualify as denes, and the associated macro-molecular configurations (together with the behavioral consequences of these configurations) would be defined as the corresponding benes. Recalling that a genitor is an implication, one could, if so desired, include the actual behavioral consequences of these configurations in the definition of the bene.

▪ Other segments of DNA without either an RNA or protein product are associated with yet different kinds of behavior. E.g., parts of the DNA, or various combinations thereof, which have a direct influence on mutation rates, would be definable as denes. A particularly interesting example of the latter is provided by stretches of small sequence repeats (SSR) that can induce slippage in the processes of replication, transcription, and even translation, and are accordingly sources of localized hyper-mutability (Moxon et al,[17]). Indeed, it has been argued that simple sequence repeats may equip the cell “with adjustable ‘tuning knobs’ for efficient adaptation” (King et al [18, p. 36]).

▪ Some denes are not associated with any material product. Thus, denes can be defined to refer to stretches of DNA (or collections of such stretches) that provide specific binding sites for particular proteins. For example, the promoter (sometimes referred to as a promoter gene) is classically identified as the region of DNA to which RNA polymerase binds before initiating the transcription of DNA into RNA, and it too is obviously a dene. But the specification of binding sites is far more general, can be far more complicated, and is crucial to biological development. Such sites inform the developmental process by securing the location of molecules with particular catalytic properties in close proximity to other molecules (or other parts of the DNA molecule) with which they can either interact directly or prevent interaction with other molecules. It is in such ways that the specificity of nucleotide sequences endows the genome with sensitivity to its chemical environment—in effect, providing a bridge between the particular regions of the genome and its environment—that is necessary for informed patterns of gene expression. Of particular interest here is the micro-structure of promoters “encoding” the logic of the transcription networks for the synthesis of key developmental proteins.

**Figure 2:**
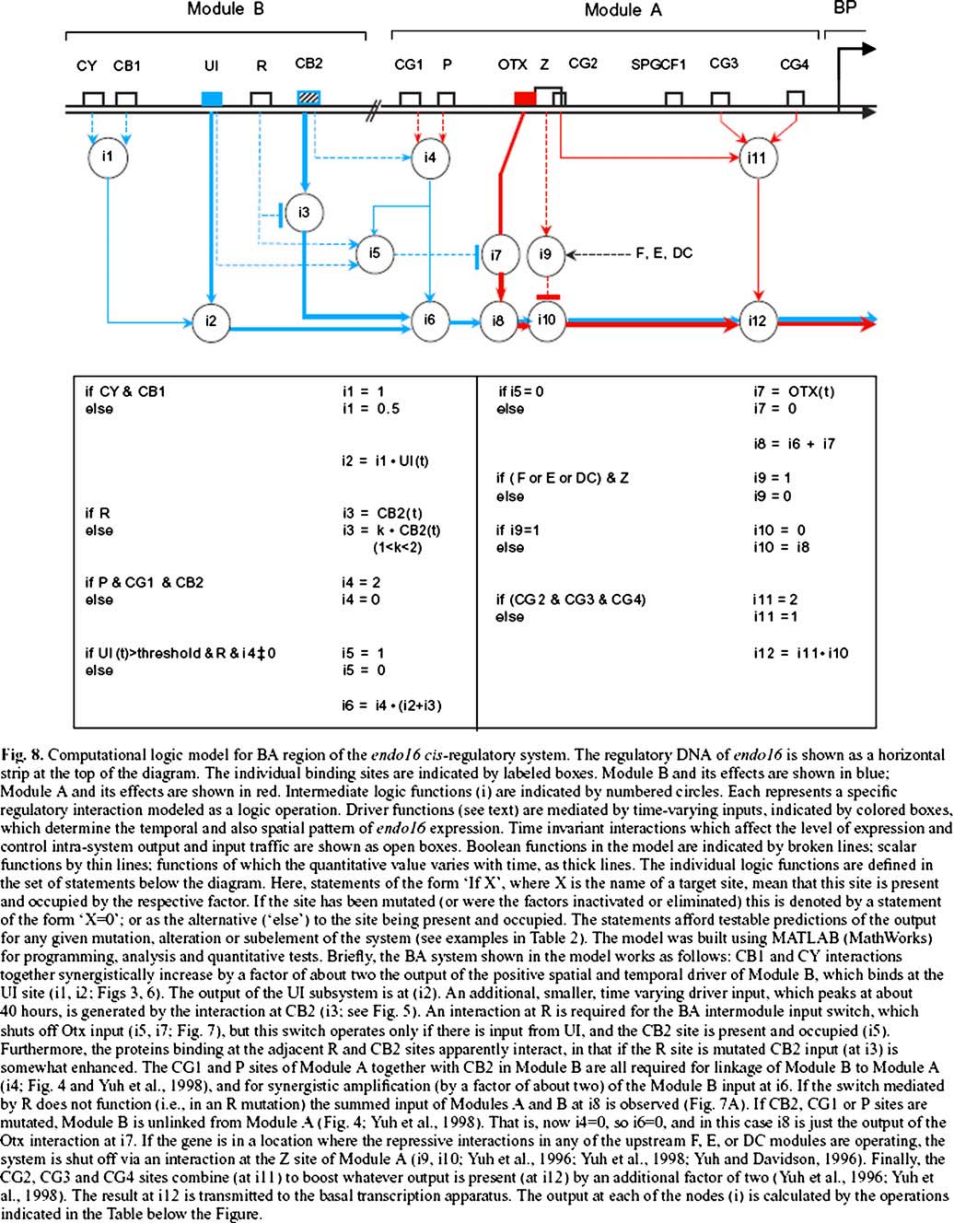
This is Fig. 8 from Yuh, Bolouri and Davidson [19], reproduced verbatim, complete with its original caption. (Reproduced with the permission of the Company of Biologists Ltd.)

▪ The best specific example of this is the model of the logic of the *endo16* genomic regulatory system in sea urchin that has been put together painstakingly, over a length and extensive period of research, by Davidson and his colleagues (see, e.g., Yuh, Bolouri and Davidson [19]). We argue that this model, its structural intricacy (dene) and behavioral complexity (bene) notwithstanding, falls naturally into the genitor framework. To that end, consider Figure 2 below, which is a reproduction of Figure 8 of that paper, caption and all. The double black horizontal line is the DNA. The annotations and rectangular markings above it give rise to a dene, which we can denote by D–*endo16promoter*. It identifies certain subsequences lying along the sea urchin's DNA at particular locations (note the slight overlapping between Z and CG2). The rest of the figure is devoted to describing the bene, call it B–*endo16*. The diagram attached to the DNA from below captures some aspects of the dynamic flow of control and information, with its various types of arrows and nodes (e.g., that the value produced at i5 is a function of two Boolean values coming from UI and R and a numeric value coming from i4, and that it produces a Boolean value that has an inhibiting effect at i7). The boxed if-then-else style of pseudo-code captures some of the logic of the circular nodes themselves; and much of the text in the caption is devoted to explaining these dynamics and adding further experimental detail (e.g., about quantities and timing, etc). Taken together, and with the addition of other figures and more detailed explanations that appear in that paper, these three types of description constitute a bene *par excellence*; they are intended to capture the dynamics of the intricate regulatory process that is part of the development of the sea urchin *S. purpuratus*, and which results from its DNA being the way it is. At the risk of sounding (or looking) pedantic, we might say that the work culminating in Figure 8 of Yuh et al [19] establishes the truth of the following genitor:
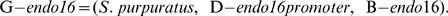



Returning to our list of examples, surely its most striking feature is its heterogeneity: At one end of the spectrum, it includes the very entities that were used not very long ago to think of as defining the gene, namely, continuous sequences of nucleotide encoding a protein (we think of this entity as the semi-classical gene). It also includes more recent ‘genomic’ conceptions of genes as exon containing entities that are not continuous, perhaps not even confined to a single chromosome, but from which proteins are constructed (we might call these genomic genes). And then, there are ncRNA genes, associated not with the production of proteins but purely with regulation. Lastly, though not finally, our list includes untranscribed (or unexpressed) collections of nucleotide sequences that acquire function in the cellular economy purely by virtue of their physical-chemical properties. We say ‘not finally’ because we assume that over time researchers will surely discover other ways in which properties of nucleotide sequences can inform function, just as biological systems, also over time, will learn other ways of making function out of sequence in the course of their evolution.

### On formalization and languages

Notice that we did not make use of a formal language in presenting the examples in the previous subsection, and that is largely because we have not yet chosen specific languages for denes and benes. But at some point we will really need to. This paper is merely intended to offer a conceptual framework to help in scientific thinking and communication, as well as to advocate its potential for both rigor and analysis. From a logical point of view, the organism in its environment can be viewed as a universe of discourse—something a logician would call a *model* or a *world*. Denes and benes are then interpreted as *predicates* that are true or false in that model, so that they would have to be written down in a formal syntax, say as formulas in some language, whose semantics yields those predicates. Once we do that, we might want to use the accepted logical notation for truth, writing O⊧D→B for the truth of a genitor (O, D, B), and read “the organism O satisfies the formula ‘D implies B’ ”. This notation also has the advantage of emphasizing the conditional nature of what is claimed about O. In fact, when talking about genitors applied to a family F of organisms, we can write F⊧D→B, and a grand genitor, true of all organisms, would be written as a validity: ⊧D→B, and read as “D always implies B”. Also, if, as mentioned earlier, the environment and mechanisms are made explicit in the bene, this notation might look like this: O⊧(D & E & M) →B, stating that the behavior B must be true in organism O if its DNA has property D, it is endowed with mechanisms M, and its environment has property E.

But what about formalizing the constituents of genitors—the denes and the benes themselves—and why do we need to do that? Isn't it enough to prepare block and arrow diagrams and some accompanying text and pseudocode, as in the sea urchin example, to clarify what we are claiming about behavior? Well, in general, no. In the good spirit of computational and systems biology, it is obvious that mathematical and algorithmic formalization of biological concepts has the advantage of—indeed is done for the purpose of—enabling computerized analysis. For genitors, the potential benefits are particularly attractive because of the ways our formulation bears on the entire agenda and discourse of biological systems, including development, behavior and evolution, and because we believe it will make it possible to clearly formulate a broad variety of results, hypotheses and research questions. These can then provide the basis for both experimental and computational analysis. For example, once we have denes and benes nailed down in adequate mathematical rigor, we can ask, and then hopefully determine algorithmically, whether certain genitors imply others; whether one group's results are consistent with another's (and if not, then what liberties in the formulation of the denes or the benes were taken to give rise to the logical inconsistency and how can they be corrected); what more needs to be done in the laboratory to establish the truth of a certain genitor, given the truth of those that have already been established; how the genitors that follow from a given set of experiments or biological data can be formulated; and so on. The capacity of mathematics, logic and computation to help in dealing with such questions cannot be overestimated.

So what should denes and benes really look like? Since they both denote truth valued functions of sequences (DNA or behavioral traces), the most general notion, the one that would encompass anything anyone would want to say (and later to analyze), would be simply Turing-computable truth-valued functions. For denes you would be allowed to say anything that can be computed from the organism's DNA, and similarly for a bene being any computable function of reactive traces. Some might be tempted to reformulate this comment as a sort of Church/Turing Thesis for biology, but we do not want to go so far, preferring to skirt the rather loaded issue of whether a cell, for example, is a true computing device in the sense of Church and Turing. At most, one might say instead that we are implying a Church/Turing thesis for meta-biology, i.e., one that holds for the prevailing scientific discourse in biology, including the processes of discussing, analyzing and modeling biological systems.

In terms of formalization, this would mean that, in principle any programming language would serve. However, just as in any other specific application area of computing, we want something tailored to the issues at hand. We want to identify more modest, and more practical, collections of properties of an organism's DNA and of its behavior; ones that seem to be appropriate to the kinds of intra-cellular mechanisms and processes that modern biology is beginning to discover.

Using terminology taken from logic and formal language theory, our earlier discussion of denes invited their formalization as the closure of atomic statements on subsequences under Boolean operators, partial order (including possible overlapping), regular expressions, and modalities. We would need probabilities and some use of natural numbers too. Many languages exist that come close to what we need here in terms of expressive power. They include variants of temporal, dynamic, or interval logics, and other kinds of calculi. We see this language as an interesting research problem to choose an appropriate one, and to endow it with a visual front-end so as to make it convenient and intuitively useful for biologists. The kinds of simple colored-chunk-along-DNA depictions shown in the figure reproduced above may not be rich enough, but they could serve as a starting point.

As to benes, here the story is different. We have to be able to talk about properties of a variety of different kinds of biological behavior and processes—the traces—on a variety of possible levels. It is clear that there will never be a single clean language for them all. The good news is that many people are working on various aspects of this, including on languages for formalizing genetic and regulatory networks and pathways, for capturing inter- and intra-cellular dynamics, and so on. In many cases either these biology-oriented languages are themselves visual and diagrammatic, or when they are not, subsequent attempts are made to endow them with visual interfaces. Still, we should emphasize the need for the bene formalism to be abstract and sufficiently high level to be able to capture dynamic reactivity in general, separately from the idiosyncratic aspects of biological detail. For this there are a variety of approaches too, including time- and probability-enriched temporal logics, algebraic and process calculi, as well as visual formalisms such as statecharts and live sequence charts.

Once this is done, many of the questions listed earlier (comparing genitors, finding inconsistencies, etc.) would fall into well-understood niches. For example, if finite-state formalisms are used, standard algorithmic verification techniques such as *model checking* (Clarke et al [20]) could be used to answer such questions. (As an aside, we might mention that there is a subtly different approach in which the system is viewed as giving rise not to a trace or traces, but to a single tree of behavior, containing as branches all possible traces. There are entire schools of thought around these two approaches, sometimes called linear-time and branching time, respectively, but we shall not get into this here.)

And while we are at it, we should also mention *executability*: A good formalism for behavior should give rise to simulatable/executable descriptions. How nice it would be if Yuh, Bolouri and Davidson [19] could have replaced the diagrams, the pseudo-code and the textual descriptions of dynamics by a full rigorous description of their complicated genitor, which would be “playable”. The user/reader would be able to make changes, remove parts, play with the DNA or with other pieces of this intricate dene-implies-bene, and behold the results [12]). We are indeed getting ahead of ourselves, but it makes sense to keep such possibilities in mind.

And we can go even further. Kapranov et al [7], for example, point to the as-of-yet poorly understood relationship between the rate (or degree) of DNA conservation between generations and the importance of the corresponding biological function—the latter being captured by the “number of functional elements that use” the relevant parts of the DNA. They also note that “a phenotype that is associated with a DNA sequence change could be a sum of the phenotypes caused by the change in all elements that share this sequence” [7, p. 420–21]. We can thus imagine defining *metrics* for denes and benes that would allow us to talk about rates of change in denes and benes, to “count” or even “sum” behaviors, and so on. All this points to the need for what we might term a *calculus* or *algebra* of genetics. The combinatorial nature of the logical/temporal structure we have suggested for genitors and their constituent denes and benes, empowered by appropriate mathematical and computational techniques, might very well be the needed starting point for this.

The wealth of possibilities is virtually limitless. The difficulties would come from the intricacy of the phenomena one tries to capture and analyze, as well as from the inherent complexity and limitations of the very notion of computation (see, e.g., Harel [21]).

## Discussion

The evolution of novel ways of making function out of sequence invites a few general remarks about the evolution of genetic novelty. In their recent book, *The Plausibility of Life* [22] Marc Kirschner and John Gerhart have squarely faced the problem that dominates so much of contemporary media attention to biology, and that is the question of whether or not natural selection is sufficient to account for the emergence of the kind of complex biological novelty that current research, not to mention ordinary perception, reveals. The neo-Darwinian credo dominant for almost a century has it that selection is the only the creative force of evolution. Kirschner and Gerhart, however, claim that the question of how “small, random genetic changes [can] be converted into complex useful innovations” has in fact so far eluded biologists, and largely because of our ignorance about the nature of biological development. To this day, they write, “the explanation for novelty has remained hidden in the organism” [22, p. 4], and it is only now that we are in a position to offer such an explanation.

The answer they propose to the question of how novelty is generated is both subtle and brilliant: Organisms, they argue, are constructed to facilitate change. Because of the loose linkages between core processes (e.g., as facilitated by the modular construction of the modern cell), organisms have a great deal of phenotypic plasticity. This plasticity facilitates phenotypic exploration, and enables organisms to adapt to changing environments. Indeed, evolution selects for such adaptability (and hence, for modularity), because the ability to adapt that modularity confers on the organism enhances survival. But crucially, such adaptability also feeds back on evolution, and it does so by changing the selective environment for genetic mutations (the Baldwin effect). In their account, the generation of genetic variants remains a random process (i.e., genetic variation is not facilitated), only phenotypic variation is facilitated, yet that alone, Kirschner and Gerhart argue, is sufficient to drive the rapid evolution of complex novel structures. Facilitated phenotypic variation suffices even to make the emergence of life plausible.

Theirs is a powerful argument, and it accords well with the structure/function duality captured by our concept of genitor; it also accords with our insistence on the need to both distinguish, and to bring together, the “what is” and the “what it does” of the organism, Yet, we do not think they go far enough. Much as we admire their book, we submit that recent research implies the possibility of at least two other routes for the evolution of novelty. Kirschner and Gerhart focus on the ways in which the experience of organisms biases the production of *phenotypic* variation, but they seem to overlook findings suggesting that the experience of organisms can bias the production of *genotypic* variation too (e.g., stress-induced mutagenesis, contingency genes, etc). Such findings suggest that genetic variation too can be facilitated—not by directly causing the production of useful mutations, but by both locally and globally biasing mutation rates, thereby increasing the probability that a useful or usable mutation might arise.

Similarly, they miss out on the implications of our changing understanding of inheritance—especially, of our growing appreciation of the importance of systems of epigenetic inheritance. If epigenetic systems of inheritance can be deployed for the inter-generational transmission of mutated denes, this would provide a far more direct vehicle for the evolution of novelty than that allowed by the Baldwin effect, important as the latter might be.

This last suggestion raises what might be the most crucial question about our proposal of a shift in focus from genes to genitors, calling attention to its greatest point of vulnerability. And that is precisely the question of how genitors are to evolve. The first condition that they must meet if they are to be evolvable is that they be inheritable. And genes, at least classically, are—whatever else they might or might not be—units of inheritance, and they are that by definition. Indeed, what clinched the locating of genes on DNA in the first place was Watson and Crick's unraveling of the wonderfully simple mechanism by which such genes could be replicated and transmitted through the generations. Even today, the great virtue of locating genes on DNA remains the fact that DNA is so conspicuously a primary and extraordinarily reliable carrier of inheritance. (Of course, that this is so is itself dependent on a complex cellular machinery for editing and repair.) Yet some of the entities that are today called genes (e.g., the mature, post-spliced, messenger encoding a protein) do not as such reside on the DNA, and it remains unclear in what sense they can be said to be inherited. Of course, the potential for forming such transcripts resides on the DNA, and *is* clearly passed on from generation to generation, but no gene coding for the particular protein in question is, *qua* gene, transmitted with that DNA. Such genes are not isolated exceptions—roughly 60% of the exons on our DNA are subject to splicing and re-sorting.

By shifting from genes as units of analysis to denes, we do not solve this problem. Even though our denes refer explicitly and exclusively to the DNA, the articulated dene cannot itself be said to be a part of the DNA. So the obvious question is: Are denes (or, more precisely, changes in denes) inherited, and if so, how? If they are, then so too could genitors be said to be inherited—at least in the sense that trait differences are said to be inherited. In other words, given the reproduction of the necessary cellular processes in the new generation, the genetic functors, the relations of implication between denes and benes, are also reproduced. Indeed, denes cannot qualify as units that evolution can either produce or make use of unless they can be shown to be inheritable. Many denes—e.g., the semi-classical gene, the sequences of nucleotides that, by their physico-chemical properties, directly inform developmental or mutational properties—are obviously inheritable. But when a dene refers to sequences of DNA that need to be articulated by the cell, or to sequences of DNA serving novel regulatory functions (e.g., the use of interference RNA in ‘gene-silencing’)—in other words, to sequences that cannot be thought of as pre-scripted in the DNA—an important question remains.

To this point, let us return to the fact that, even though DNA remains the cell's primary vehicle of inheritance, it is far from the only vehicle of inheritance. And the obvious question (already signaled above) is this: Can the prerequisites for articulating particular denes (where the specification of such articulation is not itself in the DNA) in fact be transmitted by such alternative modes of inheritance? Apparently, the answer to this question is yes. For example, several years ago, Stephen T. Smale reported the inheritability of gene silencing in the lymphocyte system [23], and more recently, Nadine Vastenhouw et al have shown “that a single episode of RNAi in the nematode *Caenorhabditis elegans* can induce transcriptional silencing effects that are inherited indefinitely in the absence of the original trigger” [24, p. 882]. The mechanism of inheritance in these systems is not yet fully understood, but it is clearly more complex than that of DNA per se, and is already known to involve the inheritance of chromatin markings. The number of examples is not large, but research into such mechanisms of inheritance has begun only recently. And the presence of even a couple of examples suggests great promise for the future of such research. Rather than attempting to bolster a concept already stretched beyond the limits it can tolerate, lines of research capable of adumbrating a conceptual framework more suitable to the current century, the century beyond the gene, clearly mark the direction in which we should be going.
